# An epidemiological examination of the subluxation construct using Hill's criteria of causation

**DOI:** 10.1186/1746-1340-17-13

**Published:** 2009-12-02

**Authors:** Timothy A Mirtz, Lon Morgan, Lawrence H Wyatt, Leon Greene

**Affiliations:** 1 University of South Dakota, Vermillion, South Dakota, USA; 2 Retired, Meridian, Idaho, USA; 3 Texas Chiropractic College, Pasadena, Texas, USA; 4 University of Kansas, Lawrence, Kansas, USA

## Abstract

**Background:**

Chiropractors claim to locate, analyze and diagnose a putative spinal lesion known as subluxation and apply the mode of spinal manipulation (adjustment) for the correction of this lesion.

**Aim:**

The purpose of this examination is to review the current evidence on the epidemiology of the subluxation construct and to evaluate the subluxation by applying epidemiologic criteria for it's significance as a causal factor.

**Methods:**

The databases of PubMed, Cinahl, and Mantis were searched for studies using the keywords subluxation, epidemiology, manipulation, dose-response, temporality, odds ratio, relative risk, biological plausibility, coherence, and analogy.

**Results:**

The criteria for causation in epidemiology are strength (strength of association), consistency, specificity, temporality (temporal sequence), dose response, experimental evidence, biological plausibility, coherence, and analogy. Applied to the subluxation all of these criteria remain for the most part unfulfilled.

**Conclusion:**

There is a significant lack of evidence to fulfill the basic criteria of causation. This lack of crucial supportive epidemiologic evidence prohibits the accurate promulgation of the chiropractic subluxation.

## Introduction

In 1843, John Stuart Mill [1] wrote a book titled *"A System of Logic: Ratiocination and Induction." *This text was used to judge causal relationships through the following means: method of agreement, method of difference, joint method of agreement and difference, method residues, and method of concomitant variation [1]. For a period of time, this approach served as the conventional wisdom regarding criteria for causation. It was not until 1965 that Sir Austin Bradford Hill [2] first summarized the epidemiologic causality criteria. Today, the criteria established by Sir Austin are applied to contemporary epidemiology as strength (strength of association), consistency, specificity, temporality (temporal sequence), dose response, experimental evidence, biological plausibility, coherence, and analogy. They form the fundamental prerequisites and assessment criteria of the cause-effect relationship [3]. This criteria have often been referred to as Hill's Criteria (Table 1). Ultimately, the strength of the evidence for concluding that there is a cause and an effect is judged by these criteria.

**Table 1:** Hill's Criteria

1	Strength
2	Consistency

3	Specificity

4	Temporal sequence

5	Dose response

6	Experimental evidence

7	Biological plausibility

8	Coherence

9	Analogy

How does Hill's Criteria apply to the chiropractic subluxation? A fundamental principle of the chiropractic profession stresses that this putative subluxation is the cause of "dis-ease," with the use of a hyphen to supposedly distinguish it from the term disease. Historically, generations of chiropractors have contended that a large percentage of all disease is caused by subluxation [4]. In fact, the early historical paradigm was that 95% of all disease (dis-ease) was due to subluxations of the spine and that the remaining 5% was caused by subluxations of the extremities, particularly the joints and feet [5]. A more contemporary view is that chiropractic health care is based on the premise that subluxations *cause *interference in the nervous system which leads to suboptimal health and symptomatic disease [6]. The Association of Chiropractic Colleges [7] paradigm statement (ACC Paradigm) suggested that "chiropractic is concerned with the preservation and restoration of health, and focuses particular attention on the subluxation." It also defined a subluxation as "a complex of functional and/or structural and/or pathological articular changes that compromise neural integrity and may influence organ system function and general health." This paradigm has been endorsed by a number of national and international organizations, including the American Chiropractic Association, the International Chiropractors' Association and the World Federation of Chiropractic [8].

Epidemiology is the study of the distribution and determinants of health-related conditions or events in defined populations and the application of this study to control health problems [9]. The basic principle of epidemiology in public health is to measure how disease is distributed in a specific population and to also determine the factors that influence or establish this distribution [10]. Epidemiology is about seeking answers to three basic questions. First, what is the problem? Second, who has the problem? Finally, why do those with the problem have it? [9] From these questions one is able to evaluate the cause of disease, measure its occurrence (prevalence and incidence), and estimate risks of contracting a disease, by determining if there are appropriate statistical associations [10]. When a statistical association between a risk factor and a disease outcome can be demonstrated, and the results of confounding and effect modification have been accounted for, Hill's Criteria, if satisfied, increase the probability that the association is causal [1].

If the chiropractic profession is to embrace the subluxation as a defining factor of health, along with claiming that intervention to correct this lesion is needed for the health of the human body, then there exists an obligation to examine the current status of subluxation against epidemiological methods and criteria. The purpose of this paper is to detail Hill's Criteria and how they may be applied to the concept of subluxation as currently embraced by the chiropractic profession.

### Defining Hill's Criteria

Hill's Criteria consists of the following: strength (strength of association), consistency, specificity, temporality (temporal sequence), dose response, experimental evidence, biological plausibility, coherence, and analogy. These nine criteria form the fundamental prerequisites and assessment of the cause-effect relationship [3]. Ultimately, the strength of the evidence for concluding that there is a cause and an effect is judged by these criteria. Hill's criteria of causality, as defined by Jenicek, [3] Rothman [11], Last [12] and Gordis [13] can be organized and defined as presented in Table 2.

**Table 2:** Definitions of Hill's Criteria

	Criteria	Definition
1	Strength	The size of the risk as measured by appropriate tests.

2	Consistency	The association is consistent when results are replicated in studies in different settings using different methods.

3	Specificity	When a single putative cause produces a specific effect.

4	Temporal sequence	Exposure always precedes the outcome.

5	Dose response	An increasing level of exposure (in amount and/or time) increases the risk.

6	Experimental evidence	The condition can be altered (prevented or ameliorated) by an appropriate experimental regimen

7	Biologic plausibility	The association agrees with currently accepted understanding of pathobiological processes.

8	Coherence	The association should be compatible with existing theory and knowledge.

9	Analogy	A finding of analogous associations between similar factors and similar diseases.

## Methods

The databases of PubMed, Cinahl, and Mantis were searched for studies using the keywords chiropractic, subluxation, epidemiology, manipulation, dose-response, temporality, odds ratio, relative risk, biological plausibility, coherence, and analogy. Combinations of the aforementioned terms were also utilized as a search strategy in order to isolate studies. Studies identified as pertinent to the purpose of this study were then accessed through the University of South Dakota's (Vermillion, South Dakota) Lomman Health Studies Library. When this search strategy failed to reveal any studies associated with the keywords each of the definitions were then isolated and additional keywords utilized. From this definition-specific method were keywords that could be placed in the search engines. For example, with the consistency criteria a keyword that was used was gender, race, and socio-economic status. Other keywords included morbidity and mortality.

## Results

Each one of Hill's Criteria was separated as a unique entity. The results of this literature review were separated by strength, consistency, specificity, temporal sequence, dose response, experimental evidence. Overall, there were no studies that demonstrated a satisfactory link of evidence to the chiropractic subluxation construct as per strength, consistency, specificity, temporal sequence, dose response, experimental evidence, biological plausibility, coherence, and analogy (Table 3). Specifically, there were no studies that found subluxation to have a relative risk or odds ratio. No studies were found that demonstrated the subluxation to be consistently found in different people of gender or race, location or even circumstance. Subluxation was not found to be specifically linked to any one disease complex. Temporal sequence studies were not noted. The subluxation was not noted in any studies related to dose response. Animal based studies that were used to satisfy the experimental evidence were limited. There were no studies that offered a biological plausibility that would isolate subluxation as a causal factor in disease. There were no studies linking the subluxation as a coherent construct and supported by generally known facts about the natural history and biology of any disease. There were no studies found that suggested the subluxation as a causal agent similar to other factually demonstrated causal agents.

## Discussion

Opinion on the current state of the epidemiology of the subluxation construct is varied. Historically, chiropractors have claimed that subluxation was directly responsible for 95% of disease. Although this percentage is not currently in evidence, there still appears to be many chiropractors that contend subluxation is still partially responsible and/or involved with disease. The opinion by chiropractors on the connection of subluxation and disease has been substantiated. Biggs et al [14] found that 68% of Canadian chiropractors believed that most diseases were caused by spinal misalignments whereas 30% of respondents agreed with the statement that the subluxation is the cause of many diseases. McDonald et al [15] reported that over 88% of their surveyed chiropractors favored retaining the term vertebral subluxation complex. Smith and Carber [16] found that over 70% of chiropractors reported that subluxation was important to their clinical decisions which guided the clinical care of the patients. McDonald et al [15] reported that a strong majority (over 75%) of their surveyed chiropractors believed that subluxation was a significant contributing factor to 50% or more of visceral disorders.

However, opinion from chiropractic scholars on an actual epidemiology of subluxation appears to be mixed. Meeker [17] suggested that clinical epidemiology concepts can be easily related to the chiropractic paradigm linking health, subluxation, and adjustment. Hawk [18] noted that combining chiropractic terminology and the terminology of public health and epidemiology one might be able to conceptualize subluxation as a risk factor for a negative health event since subluxation is not a disease in itself, but is believed to be a contributing factor to disease, illness or negative health conditions. McCoy [19,20] noted that the epidemiology of subluxation has been researched since the inception of chiropractic over 100 years ago with basic science and clinical research to further elucidate the nature of it and also suggested that research is continuing to this day. These opinions seem to suggest that an epidemiology of the subluxation is apparent and/or that subluxation can be studied using epidemiology terminology and methodology. Nonetheless, Mootz et al [21] confirmed the lack of subluxation epidemiology when they suggested that the agenda for future critical investigation for the chiropractic profession should include the vertebral subluxation complex and epidemiology relating to incidence, prevalence, and natural history. Walker [22] noted that if the subluxation as a measurable entity was valid, it would have to satisfy Hill's Criteria to reach a conclusion of causation between subluxation and visceral disease. Huijbregts [23] noted that many in the manual medicine field do not share the traditional chiropractic theoretical position on the causative or contributory role of spinal and extremity joint dysfunction in the etiology of disease. It is apparent that there is much disagreement amongst authors as to whether or not there is an epidemiology of subluxation or if subluxation can conceptually fit into a model for epidemiological study.

### Applying Hill's criteria to chiropractic subluxation

The strength of the evidence for concluding that there is a cause and an effect association between two entities must be judged by Hill's Criteria [1]. If the chiropractic subluxation is thought to be a cause, then there must logically be an effect. In other words, subluxation should be a definable and distinguishable risk factor to a person's health. While an in-depth discussion of the intricacies of risk factors is inappropriate at this time, the concept of a risk factor as it relates to human health will be explained. When a statistical association between a risk factor (e.g., putative subluxation) and a disease outcome (e.g., suboptimal health) can be demonstrated, Hill's Criteria increases the probability that the association is causal [1]. It would seem that this is an accurate assumption when the results of confounding variables and effect modification have been controlled. Table 3 summarizes the criteria of causation and the subluxation.

**Table 3:** Hill's Criteria of Causation Applied to Subluxation

	Criteria	Result
1	Strength	There were no studies that found a relative risk or odds ratio linking subluxation

2	Consistency	Subluxation has not been noted to be consistently found across any studies in different people, places, circumstances or time.

3	Specificity	There were no studies that linked disease with subluxation of any specificity. Other exposures (variables) or explanations can be given to the disease complex.

4	Temporal sequence	There were no studies suggestive of a temporal sequence linking subluxation with disease

5	Dose response	There were no studies found linking incidence of disease with magnitude of the subluxation

6	Experimental evidence	There were no consistent studies demonstrating subluxation in the animal model

7	Biological plausibility	No studies were found that offered reproducible evidence to suggest a biological plausibility of the subluxation construct.

8	Coherence	There were no studies that indicated a credible level of coherence

9	Analogy	There were no studies suggestive of a casual association via a similar agent.

### Hill's Criteria and subluxation: strength

Strength is defined as the size of the risk as measured by appropriate statistical tests [13] and is based on measurement [3]. It essentially asks the question "is the disease rate many times greater among an exposed population?" [3] In other words, the larger the association, the more likely the exposure is causing the disease [12]. Strength is measured by relative risk or odds ratios. For example, the higher the subluxation occurrence (incidence rate) in exposed subjects compared with unexposed subjects, the stronger the association [3]. In this case, subluxation would have to be found in subjects and not found in subjects. As it pertains to the subluxation there has been no evidence to suggest an incidence rate of subluxation.

### Hill's Criteria and subluxation: consistency

Consistency pertains to the association being noted consistently, across many studies in different people, places and circumstances and times [3]. Results need to be replicated in many studies.

For the chiropractic subluxation to meet this criteria it (subluxation) would have to be found repeatedly in different persons, places, times, and circumstances. In the case of a clinical condition, the subluxation would have to be consistently found with the clinical condition. To date there has not been a study that has found the subluxation in any one population (gender, race, ethnicity, age). Or that any person of gender, race, ethnicity or age is more prone to subluxation as compared to another group. Furthermore, there were no studies that determined that the subluxation is consistently present in any clinical condition.

### Hill's Criteria and subluxation: specificity

Specificity reveals that a factor (cause under study) leads to a consistent pattern of consequences [3]. In other words, a single putative cause should produce a specific effect [13]. With regards to the subluxation this is reminiscent of the "chart of effects of nerve interference" that are commonplace in chiropractic offices. This chart suggests that if a person has a subluxation or nerve interference at the fourth thoracic level then gall bladder conditions, jaundice and shingles should be made manifest. For specificity to be realized a population with any given clinical diagnosis should have a consistent and specific subluxation associated with it. For example, if a person does suffer with the diagnosis of shingles, it should specifically be noted that the fourth thoracic vertebrae is consistently associated with shingles. For the myriad of clinical conditions that exist there is not a "most common subluxation" found. Keating et al [24] noted that a review of enuresis that subluxation sites for which adjustment has been suggested to relieve enuresis ranges the entire spinal gamut.

### Hill's Criteria and subluxation: temporal sequence

Temporal sequence is the only absolutely essential criterion for a cause-effect association [12]. It asks the question "did the exposure occur before the disease began?" It (temporal sequence) must be clearly defined as the "cart is firmly behind the horse" [3]. This suggests that subluxation must always precede the clinical condition for a true cause and effect scenario to take place. If a subluxation is present for any period of time, a progression of ill effects should be observed when there is an increase in magnitude over the same period of time. However, to date there exist no evidence to support a temporal sequence of any population being exposed to subluxation and the occurrence of any clinical condition.

### Hill's Criteria and subluxation: biological plausibility

Biological plausibility refers to the coherence with the current of body of biologic knowledge [13]. The association causing an effect must agree with currently accepted understanding of pathobiological processes [12]. The criterion asks the question "does a pathophysiologic model of how the exposure could cause the disease make sense?" [3] Chiropractors often introduce subluxation as a causal factor for human disease by implicating the nervous system as the source of many health problems. No studies were found that offered reproducible evidence that the subluxation impairs nerve flow to the visceral organs and impedes health. Nor were there any studies that suggested that manipulation of subluxation removes nerve interference, restores nerve flow to the organ or eliminates nerve interference as to affect organ health.

### Hill's Criteria and subluxation: dose-response

Dose-response is defined as an increasing level of exposure (in amount and/or time) increases the risk [12]. Dose-response can be approached by asking the question "does the association show a dose response effect i.e. does the more exposure a group of people has proportionately increases the frequency of disease experienced?" [3] This criterion is an extension of the strength criterion and is based on measurement [3]. It can be explained as the measurement of the relationship amount of exposure in duration, intensity, quality and the size of the impact. The subluxation construct has yet to be defined in terms of exposure and response. Dose-response can be explained in two ways: toxicity and therapeutic. First, is the amount of exposure for any given thing enough to create pathology. Secondly, how much treatment is needed for elimination of the exposure. From the therapeutic viewpoint if a person is said to have subluxation then a certain amount of spinal manipulation should show a response, in some form, by the human body. If subluxation is a cause of any disease then logically enough spinal manipulation that is given to a patient/set of patients should show some type of response statistically that eliminates the cause. The toxicity view is that if subluxation is inherently the cause of pathology then how much subluxation in duration, intensity, quality and size of the impact is sufficient to create disease.

There was no evidence suggestive that there should be more manipulation of subluxation versus no manipulation, or even versus a few manipulations. Studies by Haas et al [25,26] on dose-response on chronic cervicogenic headache and chronic low back pain did not make mention of the subluxation construct.

### Hill's Criteria and subluxation: experimental evidence

Experimental evidence is defined as the condition can be altered (prevented or ameliorated) by an appropriate experimental regimen [12]. Rothman [11] argued that, as a criterion, experimental evidence cannot be applied to all settings. As per the subluxation, experimental evidence is lacking. However, the one area in which the experimental evidence criterion can be applied to is in the animal model. For the subluxation construct the question becomes "has the subluxation caused any pathological state in the animal model?" As well, the suggestion is made that a subluxation produced or observed in the animal model does create the environment for impaired health or lack of nerve flow for impaired end organ function. With the chiropractic construct of subluxation animal research is lacking. The only studies found pertaining to subluxation and animal experiments was DeBoer and Hansen's [27] and Henderson et al [28] work. These studies failed to isolate the subluxation as a quantifiable lesion. Cramer et al [29] noted that while animal studies are both informative and provocative the small number of studies is inadequate as an evidence base.

### Hill's Criteria and subluxation: coherence

Coherence is the association should be compatible with existing theory and knowledge [12]. The criterion essentially asks the question "is the association supported by generally known facts about the natural history and biology of the disease?" [3] To qualify as a study to match the coherence criterion a study would have to have been performed that would have suggested subluxation as a causal factor of disease and would have to be implicated in a certain disease or pathological state. In other words, in the case of a specific disease would the facts of that disease support the notion that subluxation as the causal factor. No studies were found that demonstrated that nerve irritation from chiropractic subluxation at the spinal level produces suboptimal health that requires intervention.

### Hill's Criteria and subluxation: analogy

The analogy criterion looks for a disease or exposure that may have been observed that may be of similarity. It essentially asks the question if there are no other epidemiologic or experimental studies of this exposure-disease relationship, has a causal association been established for a very similar agent? [3] No studies were found that offered a consistent analogy linking subluxation with another similar disease-producing agent.

### Limitations to utilizing Hill's Criteria

Hill's Criteria do have some limitations. The only criterion of Hill's that is truly a causal criterion is temporality while suggesting that the other criteria were vague [11]. Although these criteria were never designed to be hard and fast rules they do provide essential guidelines for establishing causation [30]. Nevertheless, the criteria of Hill remain as basic principles in finding causal relationships. Henneken and Buring's criteria are better due to the incorporation of statistical concepts and de-emphasizes the weaker criterion of analogy [31]. Nevertheless, if a concept such as subuxation fails the test established by Hill's Criteria, it would seem that the application of Henneken and Buring's criteria is premature.

Phillips and Goodman [32] have noted, in relation to Hill's criteria, that statistical significance should not be mistaken for evidence of a substantial association, association does not prove causation (other evidence must be considered), precision should not be mistaken for validity (non-random errors exist), and uncertainty about whether there is a causal relationship (or even an association) is not sufficient to suggest action should not be taken. However, these same authors noted that evidence (or belief) that there is a causal relationship is not sufficient to suggest action should be taken [32].

## Conclusion

Hill's criteria are the most commonly used epidemiologic model for suggesting a causal link for any diagnostic or treatment approach. There is a significant lack of evidence in the literature to fulfill Hill's criteria of causation with regards to the chiropractic subluxation. No supportive evidence is found for the chiropractic subluxation being associated with any disease process or of creating suboptimal health conditions requiring intervention. Regardless of popular appeal this leaves the subluxation construct in the realm of unsupported speculation. This lack of supportive evidence suggests the subluxation construct has no valid clinical applicability.

## Competing interests

The authors declare that they have no competing interests.

## Authors' contributions

TM and LM conceived the idea for the original manuscript. TM and LM and LG had initial discussions about this paper. After multiple distributions between all authors TM wrote the initial manuscripts with LM, LW and LG undertaking revisions of the work product. LM, LW, and LG offered editorial support. All authors approved the final manuscript.
